# N-mixture models with camera trap imagery produce accurate abundance estimates of ungulates

**DOI:** 10.1038/s41598-024-83011-4

**Published:** 2024-12-28

**Authors:** Grant M. Harris, David R. Stewart, Matthew J. Butler, Eric M. Rominger, Caitlin Q. Ruhl, Daniel T. McDonald, Paige M. Schmidt

**Affiliations:** 1 United States Fish and Wildlife Service, Albuquerque, NM USA; 2 New Mexico Department of Game and Fish (Retired), Santa Fe, NM USA; 3 New Mexico Department of Game and Fish, Santa Fe, NM USA; 4 United States Fish and Wildlife Service, Indiahoma, OK USA; 5 United States Fish and Wildlife Service, Tulsa, OK USA

**Keywords:** Conservation biology, Population dynamics

## Abstract

Abundance estimates inform ungulate management and recovery efforts. Yet effective and affordable estimation techniques remain absent for most ungulates lacking identifiable marks and inhabiting rugged or highly vegetated terrain. Methods using N-mixture models with camera trap imagery form an appealing solution but remain unvalidated. We assess this method using populations of desert bighorn sheep (DBS; *Ovis canadensis*) in New Mexico, USA, plus bison (*Bison bison bison*) and Texas longhorn cattle (*Bos taurus taurus*) in Oklahoma, USA, by calculating and comparing abundance estimates to censused values. We parsed data by 3 and 7-day intervals, using images filtered or unfiltered, and collected with motion detection or timed camera settings. We employed priors informed by subject matter experts (SME) and calculated using detection-nondetection methods. Abundance estimates from filtered images captured by motion detection in 3-day intervals included the censused value across all seasons for adult DBS, rams and ewes, indicating “best practices”. This “best practices” method also captured censused values for population estimates of bison (detection-nondetection) and cattle with both priors. Our assessment validates the use of N-mixture with camera trap imagery, while presenting sampling approaches, data handling procedures and model calibration to estimate wildlife population sizes more appropriately and accurately.

**Supplementary Information:**

The online version contains supplementary material available at 10.1038/s41598-024-83011-4.

## Introduction

Population sizes for many ungulates threatened with extinction or harvested for sport remain unknown or poorly defined^1^. Such animals frequently inhabit remote, rugged or heavily vegetated terrain, lack unique markings (e.g., spots or stripes), or occur in areas with untenable logistics (i.e., restricted airspace), rendering conventional abundance surveys unsuitable (e.g., aerial surveying, use of mark-capture-recapture)^1,2^. This situation impedes information gain and conservation actions to better assess, manage and secure ungulate populations, especially given the accelerating threats wrought by climate change^1,3^.

An obvious solution is generating an affordable, practical, and accurate survey approach that resolves these issues. A promising method is coupling N-mixture modeling with images acquired from camera traps^4^. The procedure is affordable and rather simple, by establishing relatively inexpensive camera traps within an appropriate sampling design, sorting imagery, identifying and counting animals, and analyzing the repeated counts within the N-mixture process. Scientists are already using this method to estimate species abundances^5–7^.

A missing step is ensuring that this procedure produces accurate estimates in real-world applications. If so, the resulting abundance estimates would vastly improve the conservation and management prospects for many ungulate species. If not, then conservation and management programs could be misled^8,9^.

We assess the application of N-mixture and camera trap imagery to validate if this method produces accurate abundance estimates of ungulate populations, thereby filling this conservation void. Assessing and validating a technique to estimate abundance requires comparing the resulting estimates to a known value. Therefore, we used N-mixture and imagery from camera traps to estimate the population sizes of wildlife populations and related these estimates to a census.

**Fig. 1:**
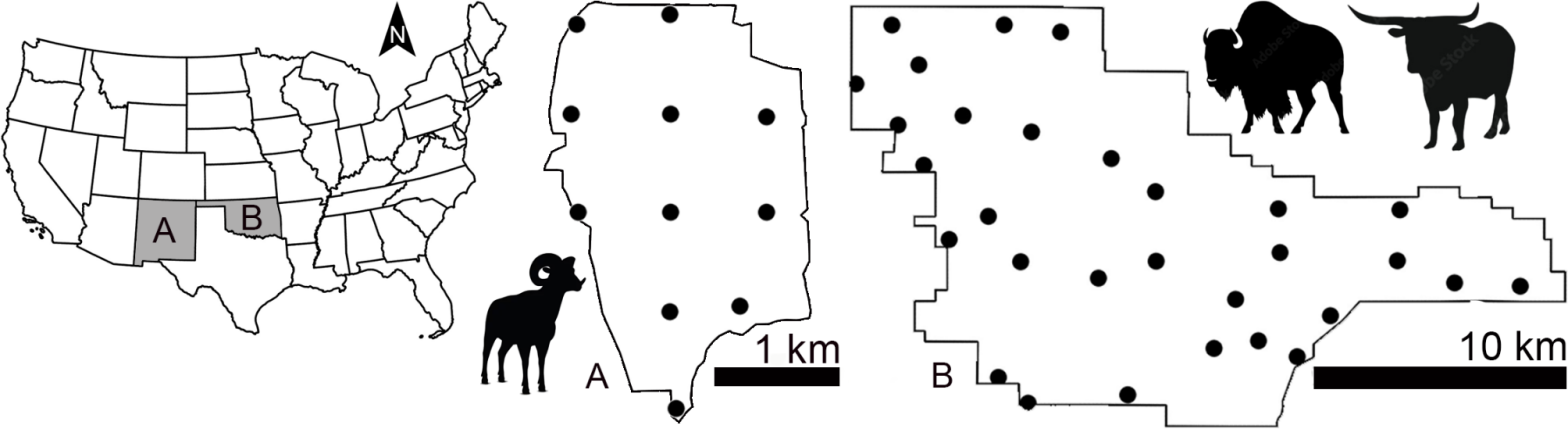
Location and boundary of study areas for estimating ungulate abundances, including a captive, wild population of desert bighorn sheep at Red Rock, New Mexico, USA (A) along with captive and wild populations of bison and Texas longhorn cattle inhabiting Wichita Mountains Wildlife Refuge in Oklahoma, USA (B) using N-Mixture modeling and images from camera traps. Circles indicate locations of camera traps, spaced ~ 800 m apart using a grid with random start at Red Rock (6.2 km^2^) and randomly located 9871.3 m apart (average) at Wichita Mountains Wildlife Refuge (243 km^2^) within mixed grass prairie and oak-juniper woodlands.

We began by estimating the abundance of a fenced, wild population of desert bighorn sheep (DBS; *Ovis canadensis*) in New Mexico, USA (Fig. 1). This population is managed by the New Mexico Department of Game and Fish (NMDGF) and censused every spring^2^. We knew the censused abundance before conducting analyses^2^. At issue was identifying the best practices for predicting that abundance.

We used the procedures learned from this analysis to inform additional assessments and validation of abundance estimation for wild populations of bison (*Bison bison bison*) and Texas longhorn cattle (cattle; *Bos taurus taurus*) inhabiting Wichita Mountains Wildlife Refuge (WMWR), in Oklahoma, USA (Fig. 1). These populations are managed by the United States Fish and Wildlife Service and annually censused. For bison and cattle, the censused abundances were provided after conducting the analyses. Here we tested if the same “best practices” identified for DBS would apply. This assessment would be telling, as these species differ in habitats used (desert vs. grassland), natural histories, densities, and the spacing of camera traps used for sampling them.

Procedurally, we examined various modeling scenarios using timed and motion activated camera traps, analyzed imagery data raw or filtered, and subset imagery by different sampling periods. Our results validate the method, while offering sampling approaches, data handling procedures and calibration of N-mixture models for helping other scientists analyze camera trap imagery using N-mixture appropriately and confidently.

N-mixture models can use Bayesian and frequentist approaches. The Bayesian method specifies a prior representing the belief of different parameter values, such as the probability of detecting an animal at a sampling site (*p*)^10^. Typically, this prior is modeled using a beta distribution (Beta(*a*,* b*), with *a* representing the shape and *b* representing the scale parameters)^10^. Priors can be defined with improper priors (reference priors), such as Beta(1,1) and Beta(0.5,0.5), using data from previous studies, or by canvasing subject matter experts (SME)^6,7,10–14^. The selection of a prior should be informed, justifiable, and carefully chosen, as the combination of the prior and data builds the final abundance estimate^10^. In our study, we utilized two priors. One prior is informed by SME. We calculated the second prior by analyzing the camera trap imagery using a detection-nondetection approach within an occupancy framework.

Previously, we analyzed this dataset of DBS imagery for validating the use of distance sampling with camera traps for abundance estimation^2^. If N-mixture models produce accurate estimates of abundance, then it would further reduce sampling logistics and project costs by eliminating distance measures.

## Results

**Fig. 2:**
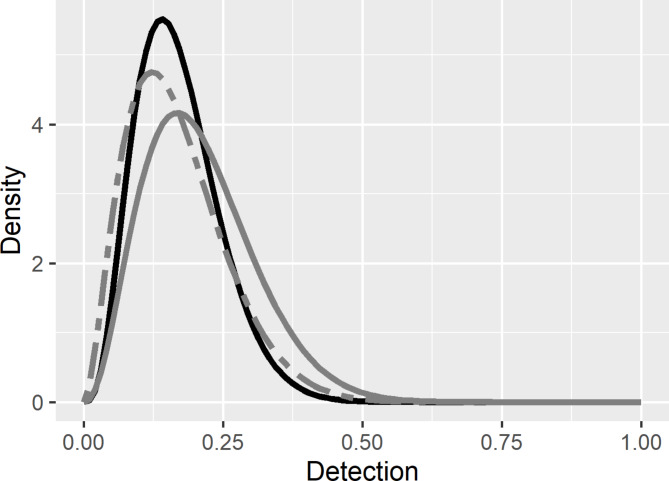
Plot describing the priors used for estimating the abundances of a desert bighorn sheep population at Red Rock, NM, USA, using N-Mixture modeling and imagery from camera traps. One prior relied on subject matter expert (SME) solicitation (black line). The second set of priors were built using detection-nondetection calculations, with imagery data parsed by 3-day (gray lines) and 7-day repeated intervals (gray dashed lines).

**Fig. 3:**
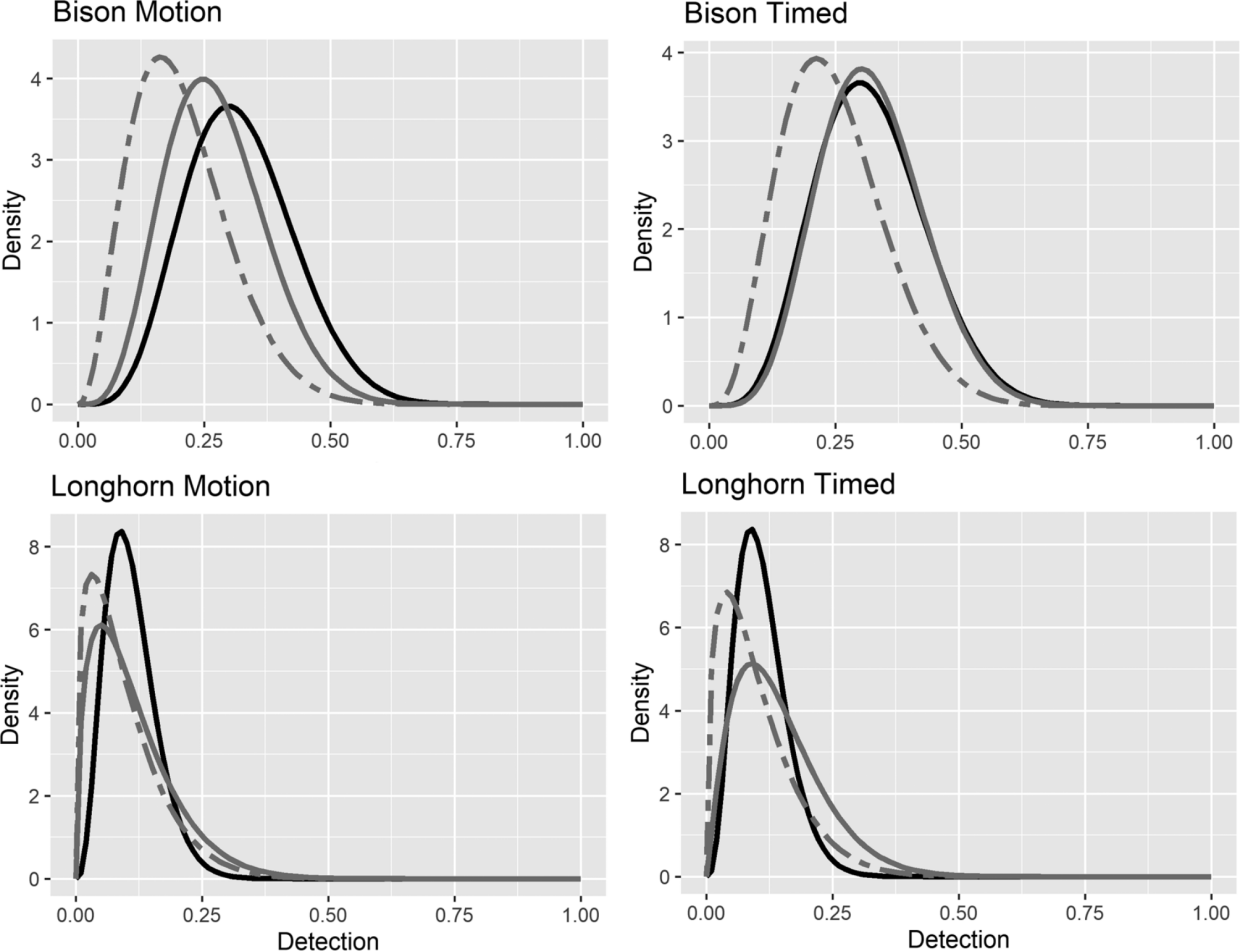
Plots describing the priors used for estimating the abundances of bison and Texas longhorn cattle populations within Wichita Mountains Wildlife Refuge, OK, USA, using N-mixture modeling and imagery from paired camera traps established in randomly located fenced exclosures (*N* = 30). One set of camera traps used motion activation and the second set of camera traps captured an image every 5 min (timed). We used priors based on subject matter expert (SME) solicitation (black lines). A second set of priors were built from detection-nondetection calculations, with data parsed by 3-day (gray lines) and 7-day intervals (gray dashed lines).

We used N-mixture modeling and imagery from camera traps to estimate the population sizes of 3 wild ungulates and compared these estimates to censused population values. Our analyses evaluated scenarios using unfiltered and filtered data with 3-day and 7-day sampling intervals. All models incorporated a prior describing the probability of a camera trap detecting a target species within the study area (*p*). We generated priors by soliciting subject matter experts (SME; Figs. 2 and 3; Table 1). For DBS, we acquired 4 SME responses to calculate a mode detection (*p*) = 0.14 with a maximum value of *p* = 0.23 having 80% confidence. At WMWR, we obtained 6 SME responses, calculating a mode and maximum *p* for bison of 0.30 and 0.41, and 0.09 and 0.15 for cattle, both with 80% confidence. We used these values to calculate the SME priors using a Beta distribution fitting method often referred to as the BetaBuster technique (Figs. 2 and 3; Table 1)^10^. We generated a second set of priors using detection-nondetection calculations (Figs. 2 and 3; Table 1).

**Table 1:** Priors used for estimating the abundances of wild, captive populations of desert bighorn sheep (DBS) at Red Rock, New Mexico, USA, plus bison and Texas longhorn cattle inhabiting Wichita Mountains Wildlife Refuge, Oklahoma, USA.

Species	Prior	Interval	Setting	a	b
DBS	SME	3 & 7	Grid	4.1	20.2
DBS	DND	3	Grid	3.4	12.9
DBS	DND	7	Grid	2.8	13.9
Bison	SME	3 & 7	Motion & Timed	5.8	12.4
Bison	DND	3	Motion	5.3	14.1
Bison	DND	3	Timed	6.4	13.5
Bison	DND	7	Motion	3.4	13.3
Bison	DND	7	Timed	4.2	12.9
Longhorn	SME	3 & 7	Motion & Timed	4.1	33.3
Longhorn	DND	3	Motion	1.6	12.6
Longhorn	DND	3	Timed	2.2	13.2
Longhorn	DND	7	Motion	1.4	13.4
Longhorn	DND	7	Timed	1.5	13.4

Priors were generated using subject matter expert (SME) solicitation and calculated using detection-nondetection analysis (DND). Data were parsed by 3 and 7-day intervals. For DBS, cameras were established in 800 m spaced grids with a random start (*N* = 11). For bison and longhorn, camera traps were established in randomly located fenced exclosures, with one set of camera traps capturing images using motion sensors, while the other set acquired an image every 5 min (timed; *N* = 30 exclosures). The priors used a Beta distribution with alpha and beta values indicated. Within each category, these priors were employed for filtered and unfiltered data.

**Fig. 4:**
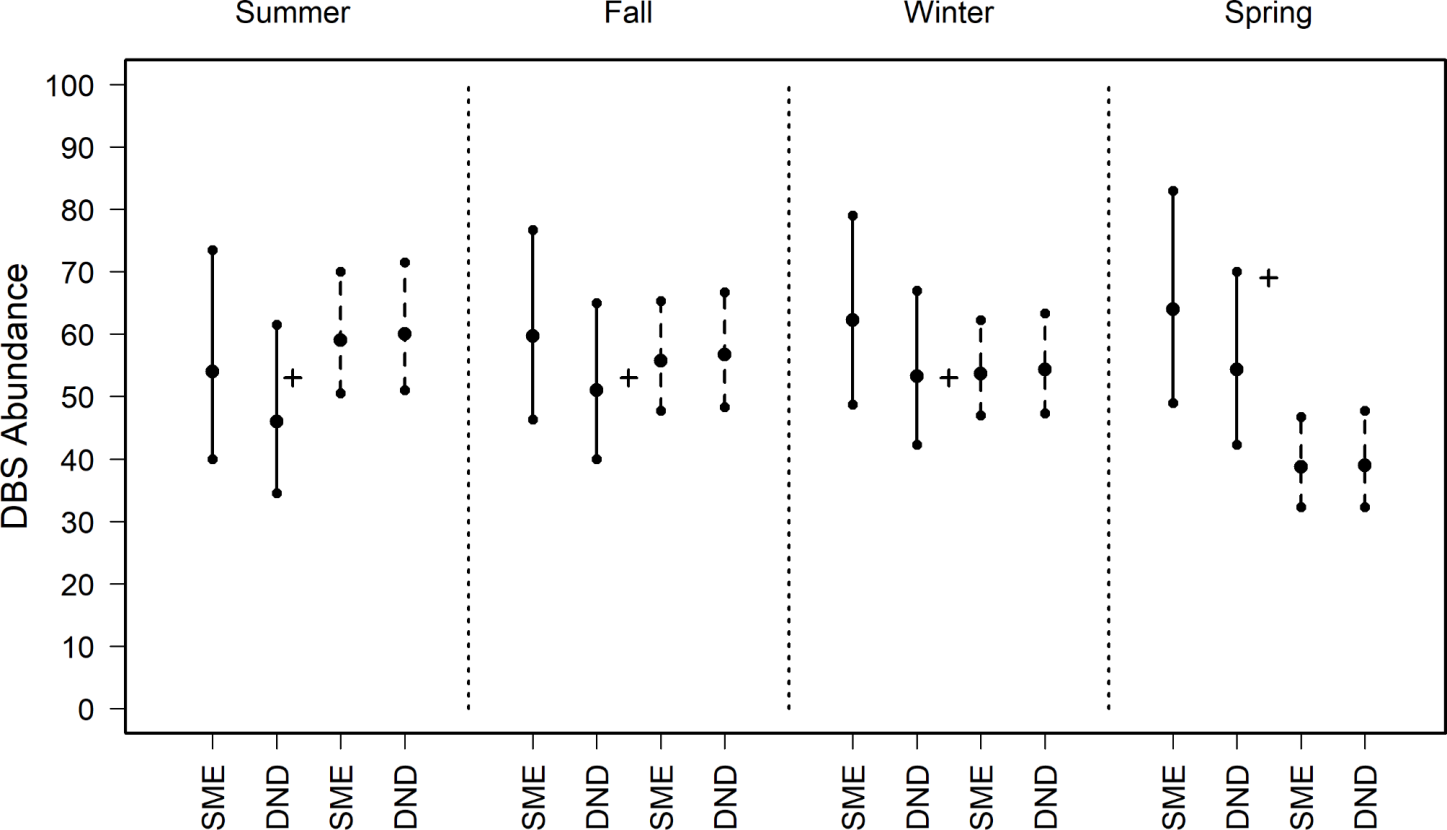
Abundance estimates produced using N-mixture modeling and camera trap images from a wild, captive population of adult desert bighorn sheep (DBS > 1.5 years; ewes, rams and adults of unknown sex) at Red Rock, NM, USA. Analyses employed priors based on subject matter experts (SME) or calculated with detection-nondetection analyses (DND). Empirical data were parsed using 3-day (solid lines) and 7-day (dashed lines) intervals, with data filtered to obtain the maximum count of adult sheep observed in visitation events separated by 1 h. For each scenario, we reported median estimates within four seasons, with 95% lower and upper credibility intervals. Seasons include Summer (June–July 2017), Autumn (August–October 2017), Winter (November 2017–January 2018) and Spring (February–April 2018). The true number of adult DBS was determined by a ground census, with 53 adults reported in May 2017 and 69 adults in May 2018 (black plus).

We estimated the number of adult DBS (adult ewes and rams > 1.5 years), for each sampling design, season, and dataset (Fig. 4; Supplementary Table 1). The censused count of desert bighorn sheep was 53 adults in May 2017 and 69 adults in May 2018.

Results from analyses using filtered data parsed to 3-day intervals contained the censused number of DBS within the credibility intervals for all seasons, with the SME and detection-nondetection prior (Fig. 4). Analyses using unfiltered data with 3-day intervals failed to predict truth in spring with both priors, in summer with the SME prior and winter with the detection-nondetection prior (Supplementary Table 1). Estimates generated with filtered data parsed by 7 days failed to capture truth in spring with both priors (Fig. 4). Abundance estimates from unfiltered data occurring in a 7-day interval never included the censused value (Supplementary Table 1).

We focused results on filtered data, as results from unfiltered data produced poor estimates (Supplementary Table 1) and unfiltered data violates N-mixture assumptions by failing to ensure independent counts^4^. We estimated the abundances of rams and ewes based on the adult estimates and the proportion of rams and ewes in the population. Across all months, the average proportion of rams was 0.45 (SD 0.11) for 3-day and 0.43 (SD 0.14) for 7-day filtered data. For ewes the mean proportion was 0.55 (SD 0.11) with 3-day data and 0.57 (SD 0.14) for 7-day filtered data.

**Fig. 5:**
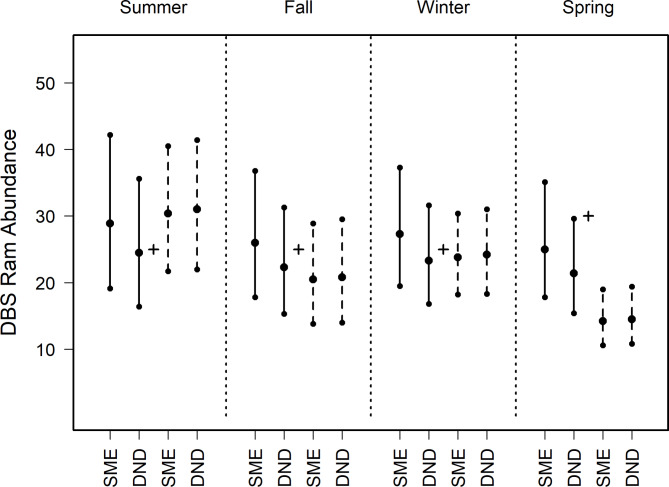
Abundance estimates of desert bighorn rams produced using N-mixture modeling and imagery from 11 camera traps for a wild, captive population (animals > 1.5 years) at Red Rock, New Mexico, USA. Analyses employed priors based on subject matter experts (SME) or calculated with detection-nondetection analyses (DND). Empirical data were filtered and parsed using 3-day (solid lines) and 7-day (dashed lines) intervals. For each scenario, we reported median estimates within four seasons, with 95% lower and upper credibility intervals. Seasons include Summer (June–July 2017), Autumn (August–October 2017), Winter (November 2017–January 2018) and Spring (February–April 2018). The true number of rams were determined by a ground census, with 25 reported in May 2017 and 30 in May 2018 (black plus).

**Fig. 6:**
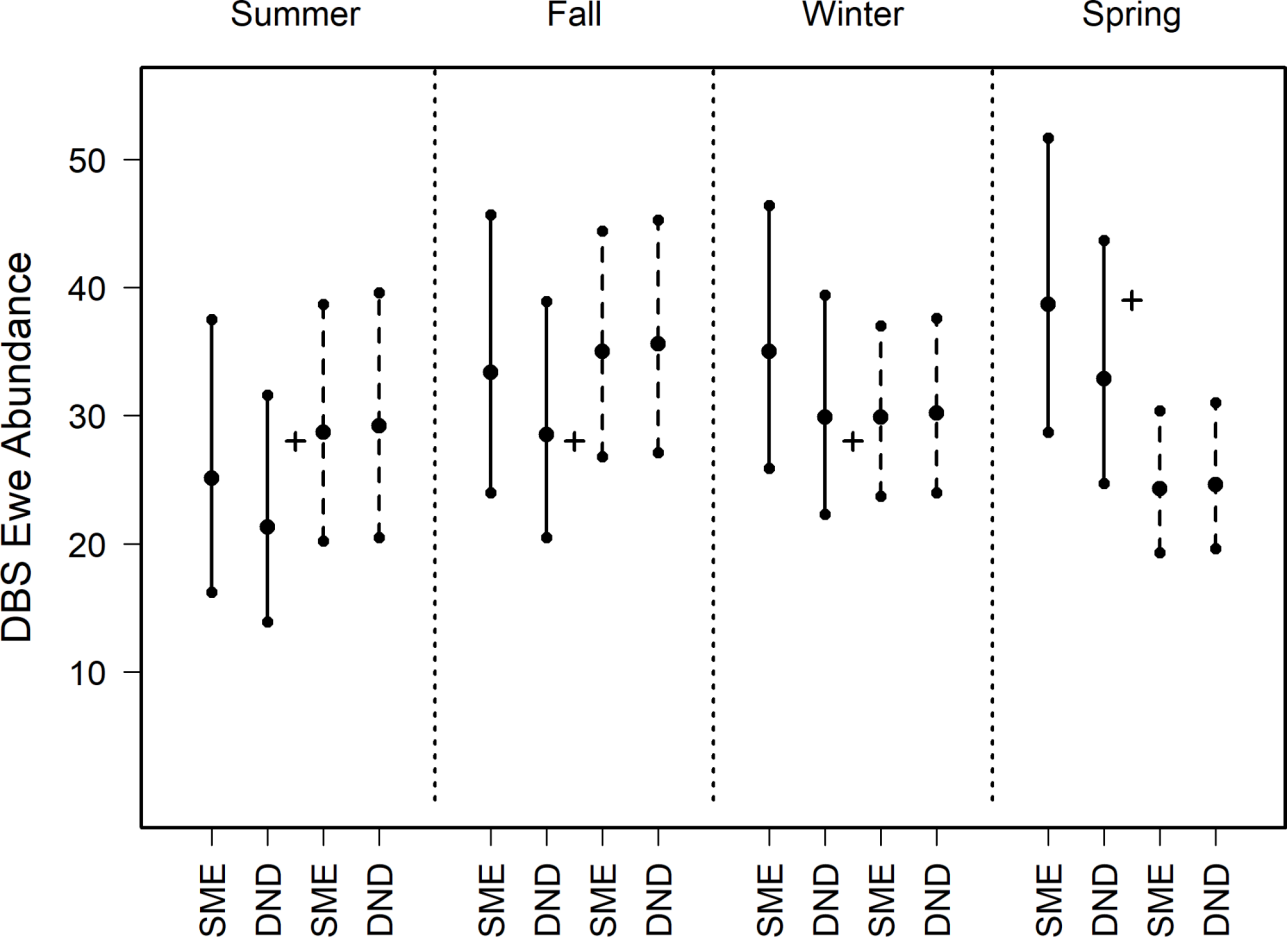
Abundance estimates of adult desert bighorn ewes produced using N-mixture modeling and imagery from 11 camera traps for a wild, captive population (animals > 1.5 years) at Red Rock, New Mexico, USA. Analyses employed priors based on subject matter experts (SME) or calculated with detection-nondetection analyses (DND). Empirical data were filtered and parsed using 3-day (solid lines) and 7-day (dashed lines) intervals. For each scenario, we reported median estimates within four seasons, with 95% lower and upper credibility intervals. Seasons include Summer (June–July 2017), Autumn (August–October 2017), Winter (November 2017–January 2018) and Spring (February–April 2018). The true number of ewes were determined by a ground census, with 28 reported in May 2017 and 39 in May 2018 (black plus).

For rams and ewes, all estimates using 3-day data with both priors contained truth (Figs. 5 and 6, Supplementary Table 2). For rams and ewes, estimates using data aggregated to 7-day intervals were consistent between priors, capturing truth in all seasons except spring (Figs. 5 and 6, Supplementary Table 2).

Census counts for young sheep included lambs and females ≤ 1.5 years, but not males ≤ 1.5 years, as they are often mistaken for ewes^8^. Across all months, the young: adult sheep ratio was 0.36 (SD 0.15) for the 3-day intervals and 0.34 (SD 0.13) for 7-day intervals with filtered data. The censused ratio was 0.53 in May 2017 and 0.51 in May 2018. None of the estimates captured the censused value within the credibility intervals (Supplementary Table 3).

The N-mixture analyses using 3-day, filtered imagery consistently produced accurate estimates of abundance for adult DBS. Therefore, we used these procedures to inform sampling and analyses for bison and cattle at WMWR. We anticipated that the “best practices” procedures revealed from the DBS analyses would also generate accurate abundance estimates for these ungulate populations.

**Fig. 7:**
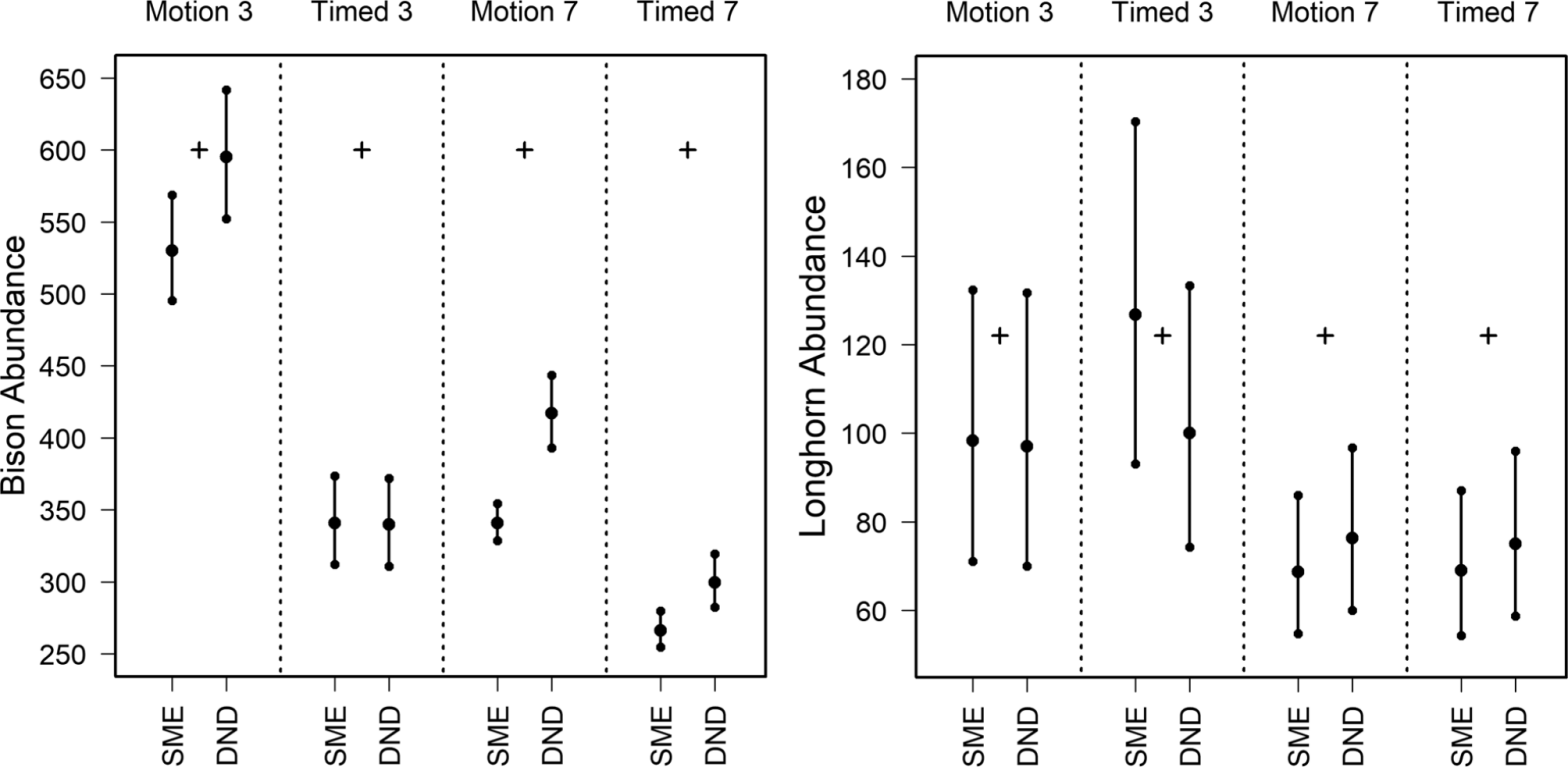
Abundance estimates generated by N-mixture modeling for captive populations of wild bison and Texas longhorn cattle inhabiting Wichita Mountains Wildlife Refuge (Oklahoma, USA). These estimates are generated from imagery acquired by motion detected camera traps (motion, *N* = 30) and cameras timed to capture an image every 5 min (timed, *N* = 30). Analyses employed priors based on subject matter experts (SME) or calculated with detection-nondetection analyses (DND). Empirical data were filtered and parsed using 3-day and 7-day intervals. These abundance estimates occur during the winter season, representing average estimates from November 2019 through January 2020. The true number of bison and cattle were determined by census, with 600 bison and 122 cattle reported in autumn 2019 (black plus).

At WMWR, we analyzed data acquired from camera traps using motion and timed settings during winter (average of November, December and January estimates; Fig. 7, Supplementary Table 4). The census count of bison is 600 animals. We estimated a median of 530.0 bison (LCL 495.3, UCL 568.7) with the SME prior and a median of 595.3 (LCL 552.3; UCL 641.7) with the detection-nondetection prior, using filtered data (3-day interval) acquired by motion detected cameras (Fig. 7, Supplementary Table 4). The true number of cattle is 122. With filtered data (3-day interval) acquired by motion detected cameras, we estimated a median of 98.3 (LCL 71.0; UCL 132.3) with the SME prior and a median of 97.0 (LCL 70.0; UCL 131.7) with the detection-nondetection prior (Fig. 7, Supplementary Table 4). Camera traps with timed settings estimated a median of 126.7 (LCL 93.0; UCL 170.3) with the SME prior and a median of 100.0 (LCL 74.3; UCL 133.3) with the detection-nondetection prior (Fig. 7, Supplementary Table 4). The remaining sampling designs failed to capture the census.

The N-mixture analyses produced estimates describing the detection process (*p*; Supplementary Table 5). Detection of DBS ranged from 0.16 to 0.21. Detection for bison ranged between 0.20 and 0.32, while detection for cattle ranged from 0.10 to 0.14 (Supplementary Table 5).

## Discussion

Abundance estimates remain scant for ungulates inhabiting remote, rugged or heavily vegetated terrain, for species lacking unique marks (e.g., spots or stripes) or occurring in places precluding traditional surveying techniques^1^. The solution requires generating an inexpensive, logistically simple and accurate estimation method appropriate for these circumstances.

We assessed and validated such a method. N-mixture models coupled with imagery acquired from camera traps can produce accurate abundance estimates. Procedurally, the data collection and N-mixture analyses are straightforward, logistically simple and economical, making the approach appealing for many conservation and management programs^1,5–7,15^. Future applications of this procedure can provide robust information to better manage and conserve ungulate populations either threatened with extinction or hunted for sport. As an example, biologists know the minimum abundance threshold for ensuring a viable population of threatened or harvested *Carpinae*^8^. The work herein provides the scientific community techniques for accurately estimating if and when that threshold is reached. Obtaining accurate abundance data becomes especially pertinent given the extensive anthropogenic pressures upon many ungulate populations, worsened by shifting climatic patterns and ecosystem conditions induced by a warming planet^3,16–18^.

Our assessment and validation help calibrate the proper use of N-mixture modeling for abundance estimation. We found that N-mixture analyses using filtered images parsed by 3-day intervals consistently produced accurate population estimates, with SME and detection-nondetection based priors. This approach captured the censused number of adult DBS, plus rams and ewes for all scenarios (Figs. 4, 5 and 6; Supplementary Tables 1–2). For bison and cattle, abundance predictions were comparable between priors, with the estimate from the detection-nondetection prior containing the censused value for bison, and estimates from both priors containing censused values for cattle (motion setting; Fig. 7, Supplementary Table 4). The “best practices” procedure using filtered imagery, 3-day intervals with motion settings always produced results appropriate for managing each of these ungulate populations. Unfiltered data, 7-day intervals and camera traps with timed settings produced less reliable results across the analytical designs.

The DBS census reports lambs, but camera traps collected relatively few images of lambs, biasing results low. This probably occurred because lambs are less mobile than adults, lamb counts peak in spring during the time of the census and decline thereafter as lambs have the highest mortality of any age group. The censused young: ewe ratio was 1.0 in May 2017 and 0.90 in May 2018. Our estimated young: ewe ratios averaged between 0.41 and 0.51 across all seasons and both priors using 3 day, filtered data. Our preference would be to quantify recruitment ratios as lamb: (adult female + yearling^8^) but the census does not report all yearlings. The N-mixture estimates aligned with prior results generated by distance sampling (i.e., mean of 20 young (90% CI 8.0–49.5)^2^.

Improper (reference) priors typically offer poor representations of *p*. For example, Beta(1,1) assumes *p* has an equal likelihood of being any value between 0 and 1.0, and Beta(0.5,0.5) consider values near 0 and 1.0 equally likely. We avoided using these and other improper priors, recognizing that uninformative priors produce uninformative results (preliminary analyses validated this outcome, as use of these priors failed to capture the census estimate)^10^, (Supplementary Table 6). We considered informing priors on *p* from related, published studies using camera traps for detecting ungulates. We rejected this approach, as the detection process is influenced by factors such as the sampling method, habitat types, species behavior and weather (Supplementary Table 5^9,19,20^). Applying the detection process gained from one study to another, or using the detection process acquired at the same location over time can generate inaccurate results^9,19,20^.

We built priors on *p* by canvassing SMEs familiar with these populations, but unfamiliar with these data, to capture their beliefs on the detection process before data analyses^10–14^. This prior is subjective, and SMEs may inaccurately estimate the detection process. The detection-nondetection method uses occupancy analyses and the imagery data being analyzed, to calculate the shape and scale parameters for the beta prior on *p*. In N-mixture analyses, detection probability (*p*) represents the probability that an individual of the species present at a site is detected, which in application indicates the probability of detecting the species at a site, matching the detection process used in occupancy modeling^4,6,20^. This detection-nondetection approach is objective, and more likely to capture true detection, thereby building greater analytical consistency and impartiality in the procedure of prior selection. Building the prior from data being analyzed could constrain the prior and cause model overfitting^10,11^. This outcome does not occur herein, as the detection-nondetection priors remain unconstrained (Figs. 2 and 3). The detection-nondetection parameter space for *p* spans realistic bounds, approximately 0–0.5 for DBS and bison, and 0–0.25 for cattle, similar to the SME prior (Figs. 2 and 3). Further, the priors built from SME and detection-nondetection add < 7 detections (alpha parameter) and < 34 absences (beta parameter) to the N-mixture data (Table 1). The imagery data contain > 1000 animal detections and absences. Inference on abundance comes from data, not the prior. The SME and detection-nondetection priors guide the model toward more realistic parameter estimates, avoiding implausible values.

Since animal detection can change seasonally, we explored building seasonal priors. Expecting SMEs to generate four different detection estimates within the same year became daunting, and calculating priors with detection-nondetection methods using data spanning all seasons ensured that *p* remained unconstrained.

Abundance comparisons and population trend analyses can be affected by the selected prior, for as shown, analyses using different priors produce different estimates. Using the same prior over time does not make an ideal fix, for as above, animal detection changes over time^9,19,20^. The proper solution is ensuring that the prior is informative, relevant and justifiable, by reflecting SME belief, designed from detection-nondetection methods or analogous techniques. Then analysts can examine a suite of appropriate estimates based on their knowledge of the population, to inform their conservation and management decisions. Using different priors in N-mixture analyses, as exemplified herein, provides an additional check on the identifiability of abundances^22^. As with any sampling technique, the separation between informative and uninformative estimates happens when analysts understand the technique assumptions, work intelligently and transparently to address them, and recognize the influence of their decisions on the results.

N-mixture models adhere to the assumption that no individual animals should be counted > 1 at or across different cameras within a given sampling interval^4^. This issue of avoiding duplicate counts is inherent to all applications of N-mixture for abundance estimation, irrespective of the species, location or sampling method.

Identifying and enacting procedures to avoid duplicate counts formed a crux of our study. We established sampling sites far enough apart, so individuals captured at one site were unlikely to be captured at another site within the sampling period. We filtered data at each site to remove duplicate counts of the same individuals at a site during the sampling period. We also limited the time interval of analyses to reduce the chances of individual animals being double counted at a site and across sites within the sampling period. These three approaches synergize.

We estimated the abundances of three wild animal populations inhabiting fenced areas. The density of DBS was approximately 10 animals /km^2^, with traps spaced 800 m apart. Densities for bison and cattle were 2.5 animals/km^2^ and 0.5 animals/km^2^, respectively, with traps spaced nearly 10 km apart. These species are capable of moving further than these trap spacings in a day, although they typically do not (e.g., DBS average ~ 40 m/ hour, bison average ~ 3 km/ day^23,24^). Importantly, the modeling scenario using a 3-day interval with filtered data produced estimates that included the censused value for all three species irrespective of their densities and the distances between camera traps, offering support for employing these methods when estimating abundances of other ungulate populations with N-mixture. Had double counting occurred, signals would be unrealistically high counts occurring within sampling intervals. We saw these signals in unfiltered data, especially with 7-day intervals, indicating sampling violations. Analysts must rely on their knowledge of the species and characteristics of the study area to inform their sampling design, count interval, and data filtering requirements. Most applications will have species density and movements unknown.

As the length of sampling intervals increase, the more homogeneous the counts become with fewer intervals lacking observations. Animal detection then converges to one, the abundance estimate converges to the maximum count recorded, and the resulting estimate tends to be biased low. This outcome was more evident in results using a 7-day interval with bison and cattle given their larger population sizes with estimates having greater divergence (Fig. 7).

Some studies recommend camera traps programmed with timed or video settings when data are analyzed with distance sampling^25,26^. Since motion detection sensors are more sensitive at closer distances, camera traps may collect images of animals nearer to the cameras, causing biases in distance measures^25,26^. Camera traps with timed settings could avoid the potential of skewed distance distributions^24,25^. Other studies have not encountered this issue with distance sampling and have produced accurate results using motion detection settings^2,27^. In our N-mixture analyses, camera traps programmed with motion detection and timed settings produced accurate results for cattle, but only with motion detection for bison (Fig. 7). We found that the timed cameras captured one image of a group’s visit, which could bias the group size and the resulting abundance low (Fig. 7). Since N-mixture relies on the series of repeated counts to generate abundance estimates, clearly the amount, magnitude, range, and variability of counts within an imagery series, within and across sites, influences the abundance results.

It’s tempting to use all the images collected by camera traps as an index of abundance^28^. Given the ease in which raw data can be assembled to become independent^29^ and analyzed within an N-mixture framework, biologists can avoid indices and produce a formal abundance estimate. The above holds provided establishment of appropriate sampling designs.

For N-mixture analyses, the size of a study area and the amount of variability in detection of the target species within that study area affects the number of sampling sites deployed. As above, sites must be appropriately spaced to avoid double counting. Higher variability in species distribution within the study area equates to more camera deployments, to meet the desired precision goals of the project. Precision is often represented by coefficient of variation (CV), with wildlife studies typically aiming for a CV ≤ 0.20^30^. Our previous work using distance sampling to analyze the same dataset for DBS indicated that obtaining a CV = 0.20 required 48 sampling locations in the spring and 62 sampling locations in the autumn, due to high variation in encounter rates^2^. In this study, were we to interpret our credibility intervals as confidence intervals, all N-mixture based abundance estimates for adult DBS had CV ≤ 0.18, a precision acquired with 11 sampling sites in a 6.2 km^2^ area (SME prior, 3-day filtered data; Supplementary Table 1). The CV for bison and cattle population estimates with 3-day filtered data from cameras using motion detection were approximately 0.04 and 0.18 respectively, using 30 sampling sites across 243 km^2^. In our case, the N-mixture analyses produced more precise abundance estimates with less deployment effort in comparison to distance sampling^2^.

Our work helps practitioners calibrate their N-mixture models for abundance estimation. When following the “best practices” identified herein, we validated that imagery from camera traps combined with N-mixture analyses produce accurate abundance estimates for ungulates. The approach offers one of the most logistically straightforward and affordable methods available. For surveys of wildlife populations, costs include purchasing, establishing and checking the camera traps, as well as collecting, organizing, and analyzing data using freeware like R. The use of N-mixture models and camera trap surveys could amplify the accessibility of abundance estimation for many ungulate and wildlife applications, worldwide. Wildlife biologists can now elucidate population sizes and trends more simply, affordably and confidently.

## Methods

We used N-mixture models for estimating the abundance of a captive population of desert bighorn sheep managed by NMDGF at Red Rock, NM, USA. The population is wild and unconditioned to humans^2^. The fenced facility contains mountainous, Chihuahuan desert terrain interspersed with deep drainages bordered by steep cliffs (6.2 km^2^).

NMDGF censuses this population every spring. The census relies on a ground crew of evenly spaced individuals performing a drive count, by walking a line while maintaining contact to count any DBS moving past the line to eliminate double counts. Most DBS move ahead of the line and are counted by biologists with spotting-scopes and binoculars at higher elevations.

We established 11 motion activated camera traps (Bushnell Trophy Cam) within the pen, on T-posts or existing vegetation at the centers of an 800-m grid having a random geographical start (Fig. 1)^2^. We oriented cameras to minimize sun exposure in the imagery by facing cameras north, while rotating the orientation eastward when necessary to avoid obstacles and ensure a clear field of view. Cameras were mounted at 0.9–1.2 m with declination perpendicular to the ground. Cameras recorded one image per trigger. Camera checks occurred every 6 months and SD cards were never full. All camera traps were deployed by 15 May 2017 and retrieved no earlier than 30 April 2018. Imagery of DBS were classified by classes of rams and ewes (males and females > 1.5 years old), young (≤ 1.5 years old), adults (rams, ewes and adult-sized animal with sex undiscernible, all > 1.5 years old), and unknown (undiscernible)^2^.

Abundance estimates of animals from N-Mixture models rely on the acquisition of temporally replicated counts at defined sampling sites^4^. The camera trap operates like a point count, consistently recording images of the target species. Individual animals do not need to be uniquely identifiable. The procedure assumes that the same individuals are not counted > 1 within the sampling period^4^.

We identified and enacted procedures to reduce the chances of double counting. First, we spaced camera traps far enough apart to reduce the chances of an animal moving between sites and captured > 1 within the sampling period. We then filtered data, by identifying independent visitation events. We considered that sequences of images were independent when *≥* 1 h elapsed between them. We chose 1 h because analyses of our data indicated that 95% of all image sequences lasted < 1 h (i.e., duration), and a 1-hour duration is corroborated by prior analyses (Supplementary Table 7)^29^. We then retained the maximum count of animals identified within a given image occurring within that visitation event^29^. For example, imagine a hypothetical series of 6 images taken at the following times (sequentially, from the first image): 0, 10, 40, 160, 3880, and 3900 s. The count of DBS in each image is 1, 3, 1, 1, 2, 1. The amount of time elapsed between images is 10, 30, 120, 3720, and 20 s. According to our method, this generates 2 independent visits. The duration of the first visit was 160 s with a count of 3 recorded, and the second independent visit lasted 20 s with a count of 2 recorded. This data filtering step reduces the likelihood of capturing the same individual animal in a series of camera trap imagery, at a given camera site. The filtering approach, however, could reduce the true count of individual animals occurring in an imagery series, thereby biasing abundance results low.

Next, we subset the imagery data to generate 3 and 7-day sampling intervals. For each camera site, we summed the maximum count of animals identified within each independent visitation event, occurring in that 3 or 7-day sampling interval. This sequence of 3 or 7-day counts at each site generated the repeat count data used in analyses. If an interval is too short (i.e., 1 day), it may not enable the site (i.e., camera trap) sufficient time to capture a representative set of animals located at the site. If the interval is too long, the site may record the same individuals multiple times. Any double counting of individuals violates the N-mixture assumption of double counting and deflates abundance estimates.

Within the N-Mixture model, the observed count histories represent a sequence of replicated counts of unmarked animals ($\:n$) at each site *i = 1*,*2…*,*I* indicating the number of sampling sites (i.e., camera traps) for a total of *j = 1*,*2…*,*J* replicate sampling occasions. The observed count histories (denoted as $\:{n}_{ij}$) can be defined by $\:{n}_{ij};i=1,\:2,\dots\:,I;j=1,\:2,\:\dots\:,J\:$and are regarded as a binomial outcome $\:h\left({n}_{ij}|{N}_{ij}{p}_{ij}\right)$ and conditional on the unknown total number of individuals available for capture $\:{N}_{ij}$ at the time of the survey and the probability of detecting an individual ($\:{p}_{ij}$):

$${n}_{ij}|{N}_{ij},{p}_{ij}\sim Bin\left({N}_{ij},{p}_{ij}\right)$$

We incorporated prior knowledge by consulting subject matter experts (SMEs) familiar with managing desert bighorn sheep (DBS) at the captive facility. The experts were presented with a formal scenario: “Imagine being placed sequentially at 100 random locations within the facility. At each location, you look ahead. On average, at how many of these sites would you expect to see at least one DBS within approximately 30 m?”. We also requested the maximum number of sites (i.e., expressed as max *p* = maximum number of sites / 100) where they would expect to observe DBS, along with their confidence level (*c*) in these estimates. We averaged the SME responses to determine *p*, considering this the mode of a beta distribution and solving by $\:p=\frac{a-1}{a+b-2}$^10^. Using a Beta distribution fitting method, commonly referred to as the BetaBuster technique, we calculated the parameter *b* for our analyses^10^. The value of *b* corresponds to the point on the horizontal axis where the probability density reaches the maximum detection probability (max *p*), given the specified confidence level *c*^10^. In this method, *b* is selected such that the beta distribution accurately reflects the expert-derived detection probabilities while accounting for the uncertainty in those estimates. Specifically, the parameter *c* is the cumulative probability up to the maximum detection probability, allowing us to determine the shape of the beta distribution and derive the SME priors. For the prior calculated with detection-nondetection data, we set the probability of a detection ($\:{y}_{ij}=1$) as the probability that at least one individual is observed. We modeled the detection-nondetection data as $\:{y}_{ij}\sim Bern\left({p}_{ij}\right)$, and in the case of the count data $\:{n}_{ij}=\text{0,1},2,\dots\:$. We account for the observation process by integrating the detection probability from the detection-nondetection data (Supplementary Table S8, Supplementary Table S9).

The model for estimating abundance uses a hierarchical marginal process. In turn, this process arises from the detection process, based on a log-link, Poisson probability ($\:{\lambda\:}_{ij}$). This formulation includes a random effect, where the mean count (intercept) follows a normal distribution across all sites, permitting the mean count to vary by sampling site.

We estimated abundance for each site and sampling period (N_ij_), then summed these by month, to generate a total abundance estimate of adult DBS for each month of the study. We generated seasonal estimates by averaging estimates from sequential months. The seasons included: Spring (February 2018–April 2018), Winter (November 2017–January 2018), Autumn (August 2017–October 2017), and Summer (June 2017–July 2017).

We estimated abundances of rams and ewes by fitting a beta distribution to the total count of rams and ewes recorded for each month and multiplying this value $\:q$ by the corresponding adult count. This $\:q$ represents the probability of selecting a ram (or ewe) in any given single selection, where $\:{a}_{0}$ and $\:{b}_{0}$ are the parameters of the prior, representing the initial beliefs about the distribution of $\:q$ before observing any data. In a scenario where no initial bias is assumed between the count of rams (*R*) and ewes (*E*), one could use a uniform prior (e.g., $\:Beta({a}_{0}=1,\:{b}_{0}=1)$), indicating no preference toward rams or ewes. The likelihood of observing $\:R$ rams out of $\:N=R+E$ trials (selections) given the probability $\:q$ of selecting a ram follows a binomial distribution, and the likelihood function is expressed as: $\:L\left(q\right|{a}_{0},{b}_{0})\propto\:{q}^{R}{\left(1-q\right)}^{E}$. Using the conjugate nature of the beta distribution with the binomial likelihood, the posterior distribution for *q* also follows a beta distribution. The posterior distribution is updated with the following parameters:

$$\:{a}_{post}={a}_{0}+R\:\text{a}\text{n}\text{d}\:{b}_{post}={b}_{0}+E$$

Thus, the posterior distribution is $\:Beta\left({a}_{post},{b}_{post}\right)$, which is proportional to: $\:{q}^{{a}_{post}-1}{\left(1-q\right)}^{{b}_{post}-1}$. This result represents our updated belief about the distribution of $\:q$ after observing $\:R$ rams and $\:E$ ewes. We followed the same procedure for estimating the number of ewes and young sheep. For simplification, with young sheep we used a beta distribution having *a* = young count + 1 and *b* = adult count + 1. All analyses incorporated separate counts corresponding to raw and filtered data (Supplementary Table 9, Supplementary Files 10 & Supplementary File 11).

The N-mixture code we employ allows *p* (detection) to vary by site, sampling period and length of the sampling period. The prior on *p* can be a reference prior (Beta(1,1), Beta(0.5,0.5)), a prior based on SME or detection-nondetection. The modeling code applies these priors in the same manner (Supplementary File 11).

We generated the detection-nondetection priors using 500,000 iterations with 50,000 burn in and 3 chains (no thinning). We evaluated all models using 3 chains, 30,000 iterations with 10,000 iterations as burn in (no thinning). Chain convergence, or stationarity, was always evaluated using the Gelman-Rubin diagnostic statistics, combined with an examination of chain histories and the posterior density plots (all Rhat values ≤ 1.0)^31^(Supplementary File 9). All quantitative analyses were done using R and WinBugs^32,33^.

We followed the DBS procedure to estimate the abundances of a wild population of bison and Texas longhorn cattle at Wichita Mountains Wildlife Refuge in Oklahoma, USA (WMWR; 243 km^2^; Fig. 1). This fenced refuge, managed by the United States Fish and Wildlife Service, encompasses mixed grass prairie, oak-juniper woodlands, and granite mountains.

At this location, we randomly located 30 camera traps within prairie and oak-juniper woodlands on T-posts set inside fenced exclosures (to reduce camera damage) averaging 9871.3 m apart (SD = 524.5; exclosures 1.5 m diameter, with camera ~ 0.3 m from fencing). As above, cameras were oriented to minimize sun exposure and mounted 0.9–1.2 m with declination perpendicular to the ground. We set cameras by 15 July 2019 and retrieved cameras by 23 February 2020. Camera traps were checked opportunistically, and we placed “NA” in data to account for the infrequent periods of camera inactivity. We deployed two different camera traps per site, one having a motion activated sensor (Bushnell Prime Low Glow), the other timed to record an image every 5 min (Reconyx PC900 Hyperfire). We analyzed these datasets separately. We retained the 1 h duration for bison and cattle, as duration values > 1 h had minimal effect on results, since the majority of durations were < 1 h (Supplementary Table 7). Each autumn (August–early November), bison and cattle are corralled and censused. The counts from autumn 2019 indicate the true number of animals in these respective populations. We centered analyses on the winter season (November 2019–January 2020).

## Electronic Supplementary Material

Below is the link to the electronic supplementary material.

Supplementary Material 1

Supplementary Material 2

Supplementary Material 3

Supplementary Material 4

Supplementary Material 5

Supplementary Material 6

Supplementary Material 7

Supplementary Material 8

Supplementary Material 9

Supplementary Material 10

Supplementary Material 11

## Data Availability

All data generated or analysed during this study are included in this published article [and its supplementary information files].

## References

[1] Shackleton, D. M. (ed.) and the IUCN/SSC Capriane Specialist Group. Wild Sheep and Goats and Their Relatives. Status Survey and Conservation Action Plan for Caprinae. IUCN, (1997).

[2] Harris, G. M., Butler, M. J., Stewart, D. R., Rominger, E. M. & Ruhl, C. Q. Accurate population estimation of *Caprinae* using camera traps and distance sampling. *Sci. Rep.* **10** (1), 17729 (2020). 33082374 10.1038/s41598-020-73893-5 PMC7576118

[3] Magurran, A. E. et al. Long-term datasets in biodiversity research and monitoring: assessing change in ecological communities through time. *Trends Ecol. Evol.* **25**, 574–582 (2010). 20656371 10.1016/j.tree.2010.06.016

[4] Royle, J. A. N-mixture models for estimating population size from spatially replicated counts. *Biometrics* **60**, 108–115 (2004). 15032780 10.1111/j.0006-341X.2004.00142.x

[5] Keever, A. C. et al. Efficacy of N-mixture models for surveying and monitoring white-tailed deer populations. *Mammal Res.* **62**, 413–422 (2017).

[6] Belant, J. L. et al. Estimating lion abundance using N-mixture models for social species. *Sci. Rep.* **6** (1), 35920 (2016). 27786283 10.1038/srep35920 PMC5082374

[7] Kafley, H. et al. Estimating prey abundance and distribution from camera trap data using binomial mixture models. *Eur. J. Wildl. Res.* **65** (5), 65–77 (2019).

[8] Harris, G. M., Butler, M. J., Stewart, D. R. & Cain, J. W. III The abundance and persistence of Caprinae populations. *Sci. Rep.* **12** (1), 13807 (2022). 35970998 10.1038/s41598-022-17963-w PMC9378773

[9] Conroy, M. J., Harris, G., Stewart, D. R. & Butler, M. J. Evaluation of desert bighorn sheep abundance surveys, southwestern Arizona, USA. *J. Wildl. Manag*. **82** (6), 1149–1160 (2018).

[10] Christensen, R., Johnson, W., Branscum, A. & Hanson, T. E. *Bayesian ideas and data analysis: an introduction for scientists and statisticians* (CRC, 2010).

[11] Choy, S. L., O’Leary, R. & Mengersen, K. Elicitation by design in ecology: using expert opinion to inform priors for Bayesian statistical models. *Ecology* **90** (1), 265–277 (2009). 19294931 10.1890/07-1886.1

[12] Martin, T. G., Kuhnert, P. M., Mengersen, K. & Possingham, H. P. The power of expert opinion in ecological models using Bayesian methods: impact of grazing on birds. *Ecol. Appl.* **15** (1), 266–280 (2005).

[13] Murray, J. V. et al. How useful is expert opinion for predicting the distribution of a species within and beyond the region of expertise? A case study using brush-tailed rock‐wallabies *Petrogale penicillata*. *J. Appl. Ecol.* **46** (4), 842–851 (2009).

[14] Kuhnert, P. M., Martin, T. G., Mengersen, K. & Possingham, H. P. Assessing the impacts of grazing levels on bird density in woodland habitat: a Bayesian approach using expert opinion. *Environmetrics: official J. Int. Environmetrics Soc.* **16** (7), 717–747 (2005).

[15] Christensen, S. A., Farr, M. T. & Williams, D. M. Assessment and novel application of N-mixture models for aerial surveys of wildlife. *Ecosphere* **12** (8), e03725 (2021).

[16] Luo, Z., Jiang, Z. & Tang, S. Impacts of climate change on distributions and diversity of ungulates on the Tibetan Plateau. *Ecol. Appl.* **25** (1), 24–38 (2015). 26255355 10.1890/13-1499.1

[17] Wu, X. et al. Predicting the shift of threatened ungulates’ habitats with climate change in Altun Mountain National Nature Reserve of the Northwestern Qinghai-Tibetan Plateau. *Clim. Change*. **142**, 331–344 (2017).

[18] Malakoutikhah, S., Fakheran, S., Hemami, M. R., Tarkesh, M. & Senn, J. Assessing future distribution, suitability of corridors and efficiency of protected areas to conserve vulnerable ungulates under climate change. *Divers. Distrib.* **26** (10), 1383–1396 (2020).

[19] Stewart, D. R., Butler, M. J., Harris, G., Johnson, L. A. & Radke, W. R. Estimating abundance of endangered fish by eliminating bias from non-constant detectability. *Endanger. Species Res.* **32**, 187–201 (2017).

[20] MacKenzie, D. I. et al. *Occupancy estimation and modeling: inferring patterns and dynamics of species occurrence* (Elsevier, 2017).

[21] Duarte, A., Adams, M. J. & Peterson, J. T. Fitting N-mixture models to count data with unmodeled heterogeneity: Bias, diagnostics, and alternative approaches. *Ecol. Model.* **374**, 51–59 (2018).

[22] Dorazio, R. M. & Royle, J. A. Rejoinder to The Performance of Mixture Models in Heterogeneous Closed Population Capture-Recapture. *Biometrics* **61**, 874–876 (2005). 10.1111/j.1541-020X.2005.00411_1.x 16135042

[23] Meagher, M. Bison bison. *Mamm. species*. **266**, 1–8 (1986).

[24] Cain, I. I. I., Krausman, J. W., Morgart, P. R., Jansen, J. R., Pepper, M. P. & B.D. & Responses of desert bighorn sheep to removal of water sources. *Wildl. Monogr.* **171** (1), 1–32 (2008).

[25] Howe, E. J., Buckland, S. T., Després-Einspenner, M. L. & Kühl, H. S. Model selection with overdispersed distance sampling data. *Methods Ecol. Evol.* **10** (1), 38–47 (2019).

[26] Howe, E. J., Buckland, S. T., Després-Einspenner, M. L. & Kühl, H. S. Distance sampling with camera traps. *Methods Ecol. Evol.* **8**, 1558–1565 (2017).

[27] Corlatti, L., Sivieri, S., Sudolska, B., Giacomelli, S. & Pedrotti, L. A field test of unconventional camera trap distance sampling to estimate abundance of marmot populations. *Wildl. Biol.* **2020(4)**, 1–11 (2020).

[28] Kenney, A. J. et al. *Motion-sensitive cameras track population abundance changes in a boreal mammal community in southwestern Yukon, Canada*pe22564 (J. Wildlife Manage., 2024).

[29] Harris, G., Sanderson, J. G., Erz, J., Lehnen, S. E. & Butler, M. J. Weather and prey predict mammals’ visitation to water. *PLoS One*. **10** (11), e0141355 (2015). 26560518 10.1371/journal.pone.0141355 PMC4641626

[30] Williams, B. K., Nichols, J. D. & Conroy, M. J. *Analysis and Management of Animal Populations* (Academic, 2002).

[31] Gelman, A. & Rubin, D. B. Inference from iterative simulation using multiple sequences. *Stat. Sci.* **7**, 457–511 (1992).

[32] R Core Team. *R: A language and environment for statistical computing.* (R Foundation for Statistical Computing, 2023) (2023). https://www.R-project.org/ 2023.

[33] Sturtz, S., Ligges, U. & Gelman, A. R2WinBUGS: A Package for Running WinBUGS from R. *J. Stat. Softw.* **12** (3), 1–16. 10.18637/jss.v012.i03 (2005).

