# Supplementary material for: N-mixture models with camera trap imagery produce accurate abundance estimates of ungulates
Source: Sci Rep. 2024 Dec 28;14:31421. doi: 10.1038/s41598-024-83011-4 (PMC11682081; doi:10.1038/s41598-024-83011-4)
Supplement: Supplementary file 1 — Supplementary Material 1 [file 41598_2024_83011_MOESM1_ESM.docx]

Supplementary Table S1: Abundance estimates produced using N-mixture modeling and imagery from 11 camera traps (800 m grid spacing) for adult desert bighorn sheep (DBS > 1.5 years; ewes, rams and adults of unknown sex) at a captive facility in New Mexico, USA. Data were parsed using 3 and 7-day intervals, with data unfiltered, or filtered to obtain the maximum count of adult sheep observed in visitation events separated by 1 h. Analyses employed priors based on subject matter experts (SME) or calculated from these data using detection-nondetection analyses. The table includes median estimates within seasons (3-month averages), with 95% lower and upper credibility intervals (LCL, UCL), standard deviation (SD) and Monte Carlo error (MCE). Seasons include Summer (June – July 2017), Autumn (August – October 2017), Winter (November 2017 – January 2018) and Spring (February – April 2018). The true number of adult desert bighorn sheep were determined by a ground census, with 53 adults in May 2017 and 69 adults in May 2018. “DND” represents detection-nondetection.

| **Interval** | **Type** | **Prior** | **Season** | **LCL** | **Median** | **UCL** | **SD** | **MCE** |
| --- | --- | --- | --- | --- | --- | --- | --- | --- |
| 3 | Filtered | SME | Summer | 40.0 | 54.0 | 73.5 | 8.6 | 0.1 |
| 3 | Filtered | SME | Autumn | 46.3 | 59.7 | 76.7 | 7.7 | 0.1 |
| 3 | Filtered | SME | Winter | 48.7 | 62.3 | 79.0 | 7.7 | 0.1 |
| 3 | Filtered | SME | Spring | 49.0 | 64.0 | 83.0 | 8.7 | 0.1 |
| 3 | Unfiltered | SME | Summer | 53.5 | 69.5 | 90.5 | 9.4 | 0.2 |
| 3 | Unfiltered | SME | Autumn | 47.0 | 60.0 | 77.0 | 7.7 | 0.1 |
| 3 | Unfiltered | SME | Winter | 31.7 | 41.7 | 54.7 | 5.8 | 0.1 |
| 3 | Unfiltered | SME | Spring | 15.3 | 22.0 | 32.0 | 4.3 | 0.1 |
| 3 | Filtered | DND | Summer | 34.5 | 46.0 | 61.5 | 7.0 | 0.1 |
| 3 | Filtered | DND | Autumn | 40.0 | 51.0 | 65.0 | 6.4 | 0.1 |
| 3 | Filtered | DND | Winter | 42.3 | 53.3 | 67.0 | 6.4 | 0.1 |
| 3 | Filtered | DND | Spring | 42.3 | 54.3 | 70.0 | 7.1 | 0.1 |
| 3 | Unfiltered | DND | Summer | 46.5 | 59.0 | 75.0 | 7.2 | 0.1 |
| 3 | Unfiltered | DND | Autumn | 41.0 | 51.3 | 64.7 | 6.0 | 0.1 |
| 3 | Unfiltered | DND | Winter | 27.7 | 35.7 | 46.0 | 4.6 | 0.1 |
| 3 | Unfiltered | DND | Spring | 13.3 | 18.7 | 26.7 | 3.4 | 0.1 |
| 7 | Filtered | SME | Summer | 50.5 | 59.0 | 70.0 | 5.0 | 0.1 |
| 7 | Filtered | SME | Autumn | 47.7 | 55.7 | 65.3 | 4.5 | 0.1 |
| 7 | Filtered | SME | Winter | 47.0 | 53.7 | 62.3 | 4.0 | 0.1 |
| 7 | Filtered | SME | Spring | 32.3 | 38.7 | 46.7 | 3.7 | 0.1 |
| 7 | Unfiltered | SME | Summer | 36.0 | 42.5 | 50.5 | 3.7 | 0.0 |
| 7 | Unfiltered | SME | Autumn | 34.0 | 39.3 | 46.7 | 3.2 | 0.0 |
| 7 | Unfiltered | SME | Winter | 32.3 | 36.3 | 42.0 | 2.5 | 0.0 |
| 7 | Unfiltered | SME | Spring | 20.0 | 23.0 | 27.3 | 1.8 | 0.0 |
| 7 | Filtered | DND | Summer | 51.0 | 60.0 | 71.5 | 5.3 | 0.1 |
| 7 | Filtered | DND | Autumn | 48.3 | 56.7 | 66.7 | 4.7 | 0.1 |
| 7 | Filtered | DND | Winter | 47.3 | 54.3 | 63.3 | 4.1 | 0.1 |
| 7 | Filtered | DND | Spring | 32.3 | 39.0 | 47.7 | 3.8 | 0.1 |
| 7 | Unfiltered | DND | Summer | 36.0 | 42.5 | 51.5 | 4.0 | 0.1 |
| 7 | Unfiltered | DND | Autumn | 34.0 | 39.7 | 47.6 | 3.4 | 0.1 |
| 7 | Unfiltered | DND | Winter | 32.3 | 36.7 | 42.7 | 2.7 | 0.0 |
| 7 | Unfiltered | DND | Spring | 20.3 | 23.0 | 27.3 | 1.9 | 0.0 |
