# Supplementary material for: N-mixture models with camera trap imagery produce accurate abundance estimates of ungulates
Source: Sci Rep. 2024 Dec 28;14:31421. doi: 10.1038/s41598-024-83011-4 (PMC11682081; doi:10.1038/s41598-024-83011-4)
Supplement: Supplementary file 2 — Supplementary Material 2 [file 41598_2024_83011_MOESM2_ESM.docx]

Supplementary Table S2: Abundance estimates produced using N-mixture modeling and imagery from 11 camera traps (800 m grid spacing) for ram and ewe desert bighorn sheep (animals > 1.5 years) at a captive facility in New Mexico, USA. Data were parsed using 3 and 7-day intervals, with data filtered to obtain the maximum count of rams or ewes observed in visitation events separated by 1 h. Analyses employed priors based on subject matter experts (SME) or calculated from these data using detection-nondetection analyses (DND). The table includes median estimates within seasons, with 95% lower and upper credibility intervals (LCL, UCL), standard deviation (SD) and Monte Carlo error (MCE). Seasons include Summer (June – July 2017), Autumn (August – October 2017), Winter (November 2017 – January 2018) and Spring (February – April 2018). The true number of desert bighorn sheep were determined by a ground census. Counts were 25 rams and 28 ewes in May 2017 and 30 rams and 39 ewes in May 2018.

| **Class** | **Interval** | **Prior** | **Season** | **LCL** | **Median** | **UCL** | **SD** | **MCE** |
| --- | --- | --- | --- | --- | --- | --- | --- | --- |
| Ram | 3 | SME | Summer | 19.1 | 28.9 | 42.2 | 5.9 | 0.1 |
| Ram | 3 | SME | Autumn | 17.8 | 26.0 | 36.8 | 4.9 | 0.1 |
| Ram | 3 | SME | Winter | 19.5 | 27.3 | 37.3 | 4.5 | 0.1 |
| Ram | 3 | SME | Spring | 17.8 | 25.0 | 35.1 | 4.4 | 0.1 |
| Ram | 3 | DND | Summer | 16.4 | 24.5 | 35.6 | 4.9 | 0.1 |
| Ram | 3 | DND | Autumn | 15.3 | 22.3 | 31.3 | 4.1 | 0.0 |
| Ram | 3 | DND | Winter | 16.8 | 23.3 | 31.6 | 3.8 | 0.0 |
| Ram | 3 | DND | Spring | 15.3 | 21.4 | 29.5 | 3.6 | 0.0 |
| Ram | 7 | SME | Summer | 21.7 | 30.4 | 40.5 | 4.8 | 0.0 |
| Ram | 7 | SME | Autumn | 13.8 | 20.5 | 28.9 | 3.9 | 0.0 |
| Ram | 7 | SME | Winter | 18.2 | 23.8 | 30.4 | 3.1 | 0.0 |
| Ram | 7 | SME | Spring | 10.6 | 14.2 | 19.0 | 2.1 | 0.0 |
| Ram | 7 | DND | Summer | 22.0 | 31.0 | 41.4 | 5.0 | 0.0 |
| Ram | 7 | DND | Autumn | 14.0 | 20.8 | 29.5 | 4.0 | 0.0 |
| Ram | 7 | DND | Winter | 18.3 | 24.2 | 31.0 | 3.3 | 0.0 |
| Ram | 7 | DND | Spring | 10.8 | 14.5 | 19.4 | 2.2 | 0.0 |
| Ewe | 3 | SME | Summer | 16.2 | 25.1 | 37.5 | 5.4 | 0.1 |
| Ewe | 3 | SME | Autumn | 24.0 | 33.4 | 45.7 | 5.6 | 0.1 |
| Ewe | 3 | SME | Winter | 25.9 | 35.0 | 46.4 | 5.3 | 0.1 |
| Ewe | 3 | SME | Spring | 28.7 | 38.7 | 51.7 | 5.9 | 0.1 |
| Ewe | 3 | DND | Summer | 13.9 | 21.3 | 31.6 | 4.5 | 0.1 |
| Ewe | 3 | DND | Autumn | 20.5 | 28.5 | 38.9 | 4.7 | 0.1 |
| Ewe | 3 | DND | Winter | 22.3 | 29.9 | 39.4 | 4.4 | 0.1 |
| Ewe | 3 | DND | Spring | 24.7 | 32.9 | 43.7 | 4.9 | 0.1 |
| Ewe | 7 | SME | Summer | 20.2 | 28.7 | 38.7 | 4.7 | 0.0 |
| Ewe | 7 | SME | Autumn | 26.8 | 35.0 | 44.4 | 4.5 | 0.0 |
| Ewe | 7 | SME | Winter | 23.7 | 29.9 | 37.0 | 3.4 | 0.0 |
| Ewe | 7 | SME | Spring | 19.3 | 24.3 | 30.4 | 2.8 | 0.0 |
| Ewe | 7 | DND | Summer | 20.5 | 29.2 | 39.6 | 4.9 | 0.0 |
| Ewe | 7 | DND | Autumn | 27.1 | 35.6 | 45.3 | 4.7 | 0.0 |
| Ewe | 7 | DND | Winter | 24.0 | 30.2 | 37.6 | 3.5 | 0.0 |
| Ewe | 7 | DND | Spring | 19.6 | 24.6 | 31.0 | 2.9 | 0.0 |
