# Supplementary material for: N-mixture models with camera trap imagery produce accurate abundance estimates of ungulates
Source: Sci Rep. 2024 Dec 28;14:31421. doi: 10.1038/s41598-024-83011-4 (PMC11682081; doi:10.1038/s41598-024-83011-4)
Supplement: Supplementary file 3 — Supplementary Material 3 [file 41598_2024_83011_MOESM3_ESM.docx]

Supplementary Table S3: Abundance estimates produced using N-mixture modeling and imagery from 11 camera traps (800 m grid spacing) for young desert bighorn sheep (animals < 1.5 years) at a captive facility in New Mexico, USA. Data were parsed using 3 and 7-day intervals, with data filtered to obtain the maximum count of young sheep observed in visitation events separated by 1 h. Analyses employed priors based on subject matter experts (SME) or calculated from these data using detection-nondetection analyses (DND). The table includes median estimates within seasons (3-month averages), with 95% lower and upper credibility intervals (LCL, UCL), standard deviation (SD) and Monte Carlo error (MCE). Seasons include Summer (June – July 2017), Autumn (August – October 2017), Winter (November 2017 – January 2018) and Spring (February – April 2018). The true number of young desert bighorn sheep were determined by an annual ground census, which included lambs and females (< 1.5 years). The counts indicated 28 young bighorn sheep in May 2017 and 35 in May 2018.

| \| **Interval** \| **Prior** \| **Season** \| **LCL** \| **Median** \| **UCL** \| **SD** \| **MCE** \| \| --- \| --- \| --- \| --- \| --- \| --- \| --- \| --- \| \| 3 \| SME \| Summer \| 5.5 \| 10.2 \| 17.5 \| 3.1 \| 0.0 \| \| 3 \| SME \| Autumn \| 10.9 \| 16.5 \| 24.0 \| 3.4 \| 0.0 \| \| 3 \| SME \| Winter \| 10.2 \| 15.0 \| 21.7 \| 2.9 \| 0.0 \| \| 3 \| SME \| Spring \| 14.1 \| 19.7 \| 27.3 \| 3.4 \| 0.0 \| \| 3 \| DND \| Summer \| 4.7 \| 8.6 \| 14.7 \| 2.6 \| 0.0 \| \| 3 \| DND \| Autumn \| 9.3 \| 14.1 \| 20.3 \| 2.8 \| 0.0 \| \| 3 \| DND \| Winter \| 8.8 \| 12.9 \| 18.4 \| 2.5 \| 0.0 \| \| 3 \| DND \| Spring \| 12.1 \| 16.8 \| 23.0 \| 2.8 \| 0.0 \| \| 7 \| SME \| Summer \| 5.9 \| 10.3 \| 16.4 \| 2.7 \| 0.0 \| \| 7 \| SME \| Autumn \| 9.8 \| 14.9 \| 21.6 \| 3.0 \| 0.0 \| \| 7 \| SME \| Winter \| 9.5 \| 13.2 \| 17.9 \| 2.1 \| 0.0 \| \| 7 \| SME \| Spring \| 9.0 \| 12.0 \| 15.8 \| 1.8 \| 0.0 \| \| 7 \| DND \| Summer \| 6.0 \| 10.5 \| 16.8 \| 2.8 \| 0.0 \| \| 7 \| DND \| Autumn \| 10.0 \| 15.1 \| 21.9 \| 3.1 \| 0.0 \| \| 7 \| DND \| Winter \| 9.6 \| 13.4 \| 18.2 \| 2.2 \| 0.0 \| \| 7 \| DND \| Spring \| 9.1 \| 12.1 \| 16.1 \| 1.8 \| 0.0 \| |  |  |  |  |  |  |  |  |
| --- | --- | --- | --- | --- | --- | --- | --- | --- | --- | --- | --- | --- | --- | --- | --- | --- | --- | --- | --- | --- | --- | --- | --- | --- | --- | --- | --- | --- | --- | --- | --- | --- | --- | --- | --- | --- | --- | --- | --- | --- | --- | --- | --- | --- | --- | --- | --- | --- | --- | --- | --- | --- | --- | --- | --- | --- | --- | --- | --- | --- | --- | --- | --- | --- | --- | --- | --- | --- | --- | --- | --- | --- | --- | --- | --- | --- | --- | --- | --- | --- | --- | --- | --- | --- | --- | --- | --- | --- | --- | --- | --- | --- | --- | --- | --- | --- | --- | --- | --- | --- | --- | --- | --- | --- | --- | --- | --- | --- | --- | --- | --- | --- | --- | --- | --- | --- | --- | --- | --- | --- | --- | --- | --- | --- | --- | --- | --- | --- | --- | --- | --- | --- | --- | --- | --- | --- | --- | --- | --- | --- | --- | --- | --- | --- |
