# Supplementary material for: N-mixture models with camera trap imagery produce accurate abundance estimates of ungulates
Source: Sci Rep. 2024 Dec 28;14:31421. doi: 10.1038/s41598-024-83011-4 (PMC11682081; doi:10.1038/s41598-024-83011-4)
Supplement: Supplementary file 4 — Supplementary Material 4 [file 41598_2024_83011_MOESM4_ESM.docx]

Supplementary Table 4: Abundance estimates for captive populations of wild bison and Texas longhorn cattle inhabiting Wichita Mountains Wildlife Refuge (Oklahoma, USA), built from counts of animals acquired using motion detected camera traps and camera traps programmed to collect an image every 5 minutes (timed). Data were analyzed using a N-mixture modeling approach. All raw data were filtered to acquire independent imagery, by analyzing the maximum count of individual bison or longhorn occurring within a given image, for each camera, within visitation events separated by 1 h. Interval represents a 3 or 7-day repeated sums of animal counts by camera. We used priors informed by subject matter experts (SME) and calculated using detection-nondetection methods (DND). Results encompass the winter season, built by averaging estimates from November through January. Columns LCL and UCL report the upper and lower credibility intervals, with SD the standard deviation and MCE the Monte Carlo error. The censused number of animals are 122 Texas longhorn cattle and 600 bison.

| **Setting** | **Interval** | **Prior** | **Species** | **LCI** | **Median** | **UCI** | **SD** | **MCE** |
| --- | --- | --- | --- | --- | --- | --- | --- | --- |
| Motion | 3 | SME | Bison | 495.3 | 530.0 | 568.7 | 18.6 | 0.2 |
| Motion | 3 | DND | Bison | 552.3 | 595.3 | 641.7 | 22.2 | 0.3 |
| Motion | 7 | SME | Bison | 328.7 | 340.7 | 354.3 | 6.6 | 0.1 |
| Motion | 7 | DND | Bison | 393.0 | 417.0 | 443.7 | 12.9 | 0.2 |
| Motion | 3 | SME | Longhorn | 71.0 | 98.3 | 132.3 | 15.8 | 0.3 |
| Motion | 3 | DND | Longhorn | 70.0 | 97.0 | 131.7 | 15.7 | 0.3 |
| Motion | 7 | SME | Longhorn | 54.7 | 68.7 | 86.0 | 8.0 | 0.1 |
| Motion | 7 | DND | Longhorn | 60.0 | 76.33 | 96.67 | 9.4 | 0.2 |
| Timed | 3 | SME | Bison | 312.0 | 341.0 | 373.7 | 15.8 | 0.2 |
| Timed | 3 | DND | Bison | 310.7 | 340.0 | 372.0 | 15.7 | 0.2 |
| Timed | 7 | SME | Bison | 254.7 | 266.3 | 279.7 | 6.4 | 0.1 |
| Timed | 7 | DND | Bison | 282.3 | 299.7 | 319.3 | 9.4 | 0.1 |
| Timed | 3 | SME | Longhorn | 93.0 | 126.7 | 170.3 | 19.7 | 0.4 |
| Timed | 3 | DND | Longhorn | 74.3 | 100.0 | 133.3 | 15.0 | 0.3 |
| Timed | 7 | SME | Longhorn | 54.3 | 69.0 | 87.0 | 8.3 | 0.1 |
| Timed | 7 | DND | Longhorn | 58.7 | 75.0 | 96.0 | 9.6 | 0.2 |
