# Supplementary material for: N-mixture models with camera trap imagery produce accurate abundance estimates of ungulates
Source: Sci Rep. 2024 Dec 28;14:31421. doi: 10.1038/s41598-024-83011-4 (PMC11682081; doi:10.1038/s41598-024-83011-4)
Supplement: Supplementary file 5 — Supplementary Material 5 [file 41598_2024_83011_MOESM5_ESM.docx]

Supplementary Table S5: Detection (*p*) from N-mixture analyses calculated during the estimation of population sizes of adult desert bighorn sheep (DBS; New Mexico, USA), plus bison and Texas longhorn cattle at Wichita Mountains Wildlife Refuge (Oklahoma, USA). Data were unfiltered or filtered to acquire independent imagery, by analyzing the maximum count of individual animals occurring within a given image, for each camera, within visitation events separated by 1 h. Interval represents a 3 or 7-day repeated sums of animal counts by camera. We used priors informed by subject matter experts (SME) and calculated using detection-nondetection methods (DND). For bison and cattle, we employed motion activated trail cameras (M) and cameras timed to capture images at 5 min intervals (T).

| **Species** | **Interval** | **Type** | **Prior** | ***p*** |
| --- | --- | --- | --- | --- |
| DBS | 3 | Filtered | SME | 0.17 |
| DBS | 3 | Unfiltered | SME | 0.17 |
| DBS | 3 | Filtered | DND | 0.20 |
| DBS | 3 | Unfiltered | DND | 0.21 |
| DBS | 7 | Filtered | SME | 0.17 |
| DBS | 7 | Unfiltered | SME | 0.16 |
| DBS | 7 | Filtered | DND | 0.17 |
| DBS | 7 | Unfiltered | DND | 0.16 |
| Bison | 3 | Filtered M | SME | 0.32 |
| Bison | 3 | Filtered M | DND | 0.27 |
| Bison | 3 | Filtered T | SME | 0.32 |
| Bison | 3 | Filtered T | DND | 0.32 |
| Bison | 7 | Filtered M | SME | 0.31 |
| Bison | 7 | Filtered M | DND | 0.20 |
| Bison | 7 | Filtered T | SME | 0.31 |
| Bison | 7 | Filtered T | DND | 0.24 |
| Longhorn | 3 | Filtered M | SME | 0.11 |
| Longhorn | 3 | Filtered M | DND | 0.11 |
| Longhorn | 3 | Filtered T | SME | 0.11 |
| Longhorn | 3 | Filtered T | DND | 0.14 |
| Longhorn | 7 | Filtered M | SME | 0.11 |
| Longhorn | 7 | Filtered M | DND | 0.10 |
| Longhorn | 7 | Filtered T | SME | 0.11 |
| Longhorn | 7 | Filtered T | DND | 0.10 |
