# Supplementary material for: N-mixture models with camera trap imagery produce accurate abundance estimates of ungulates
Source: Sci Rep. 2024 Dec 28;14:31421. doi: 10.1038/s41598-024-83011-4 (PMC11682081; doi:10.1038/s41598-024-83011-4)
Supplement: Supplementary file 6 — Supplementary Material 6 [file 41598_2024_83011_MOESM6_ESM.docx]

Supplementary Table 6: Abundance estimates of desert bighorn sheep, bison and Texas longhorn cattle, using imagery data acquired by trail cameras and N-mixture analyses with reference priors Beta(1,1) and Beta(0.5,0.5). Data below represent filtered imagery with three day intervals and motion activated cameras. Models operated with 30,000 iterations with 10,000 burn in without thinning and 3 chains. The use of reference priors failed to capture the censused value (Adult desert bighorn sheep census = 69 in spring and 53 in other periods; bison = 600; Texas longhorn cattle = 122).

| Beta(1,1) |  |  |  |  |
| --- | --- | --- | --- | --- |
| Season / Species | 2.50% | median | 9.75% | Rhat |
| Fall / Adult DBS | 26.7 | 30.3 | 35.7 | 1 |
| Spring / Adult DBS | 28 | 32 | 37.7 | 1 |
| Summer / Adult DBS | 23 | 26.5 | 32 | 1 |
| Winter / Adult DBS | 28 | 31.7 | 37.3 | 1 |
| Winter / Bison | 398.7 | 422 | 448.7 | 1 |
| Winter / Texas Longhorn Cattle | 25.6 | 30.7 | 37 | 1 |
| Beta(0.5,0.5) |  |  |  |  |
| Season / Species | 2.50% | median | 9.75% | Rhat |
| Fall / Adult DBS | 26.7 | 30.3 | 35.6 | 1 |
| Spring / Adult DBS | 28 | 32 | 37.7 | 1 |
| Summer / Adult DBS | 23 | 26.5 | 32 | 1 |
| Winter / Adult DBS | 27.7 | 31.7 | 37 | 1 |
| Winter / Bison | 396.7 | 423.7 | 454.3 | 1 |
| Winter / Texas Longhorn Cattle | 24.6 | 29.3 | 36 | 1 |
