# Supplementary material for: N-mixture models with camera trap imagery produce accurate abundance estimates of ungulates
Source: Sci Rep. 2024 Dec 28;14:31421. doi: 10.1038/s41598-024-83011-4 (PMC11682081; doi:10.1038/s41598-024-83011-4)
Supplement: Supplementary file 7 — Supplementary Material 7 [file 41598_2024_83011_MOESM7_ESM.docx]

Supplementary Table S7: Duration of visitation events for desert bighorn sheep (DBS) at Red Rock, NM, USA, plus bison and Texas longhorn cattle (cattle) inhabiting Wichita Mountains Wildlife Refuge, OK, USA, captured in imagery gained by camera traps set with a motion sensor (M) or timed to record an image every 5 minutes (T). Duration represents the amount of time a visitation event lasted, calculated by quantifying the length of time for each imagery series. For example, DBS and longhorn cattle had 95% of all visitation events last < 1 h, while bison had 95% of all visitation events last 1 hour and 9.6 minutes with motion enabled cameras.

| DBS (M) | DBS (M) | Bison (M) | Bison (T) | Cattle (M) | Cattle (T) |
| --- | --- | --- | --- | --- | --- |
| Quantile | Prop. | Prop. | Prop. | Prop. | Prop. |
| 25% | 0.00 | 0.00 | 0.00 | 0.00 | 0.00 |
| 30% | 0.00 | 0.01 | 0.00 | 0.00 | 0.00 |
| 35% | 0.00 | 0.01 | 0.00 | 0.00 | 0.00 |
| 40% | 0.00 | 0.02 | 0.00 | 0.00 | 0.00 |
| 45% | 0.00 | 0.02 | 0.08 | 0.00 | 0.00 |
| 50% | 0.00 | 0.04 | 0.08 | 0.01 | 0.08 |
| 55% | 0.00 | 0.05 | 0.08 | 0.01 | 0.08 |
| 60% | 0.01 | 0.07 | 0.17 | 0.01 | 0.17 |
| 65% | 0.02 | 0.11 | 0.17 | 0.01 | 0.17 |
| 70% | 0.03 | 0.16 | 0.25 | 0.02 | 0.25 |
| 75% | 0.06 | 0.22 | 0.33 | 0.07 | 0.40 |
| 80% | 0.17 | 0.32 | 0.50 | 0.14 | 0.50 |
| 85% | 0.30 | 0.46 | 0.81 | 0.30 | 0.70 |
| 90% | 0.56 | 0.66 | 1.04 | 0.49 | 1.28 |
| 95% | 0.96 | 1.16 | 1.60 | 0.92 | 2.11 |
| 99% | 1.95 | 2.36 | 2.96 | 2.51 | 4.89 |
| 100% | 4.14 | 2.99 | 5.50 | 6.81 | 6.25 |
